# Low-Cost and Tough Pneumatic Artificial Muscle (LT-PAM)

**DOI:** 10.1016/j.ohx.2026.e00824

**Published:** 2026-08-20

**Authors:** Keisuke Naniwa, Yasuhiro Sugimoto, Daisuke Nakanishi, Yoichi Masuda

**Affiliations:** a Department of Mechanical Engineering, Faculty of Engineering, Hokkaido University of Science, 15-4-1, Maeda 7-jo, Teine, Sapporo, Hokkaido, 006-8585, Japan; b Department of Electrical and Electronic Systems Engineering, Faculty of Engineering, Osaka Institute of Technology, 5-16-1 Omiya, Asahi-ku, Osaka, 535-8585, Japan; c Department of Control Engineering, National Institute of Technology, Matsue College, 14-4 Nishi-ikuma, Matsue, Shimane 690-8518, Japan; d Department of Mechanical Engineering, The University of Osaka, 2-1 Yamadaoka, Suita, Osaka 565-0871, Japan

**Keywords:** McKibben-type actuator, Soft robotics hardware, High-load soft actuator, Crimp-based end fixation, Reproducible lab fabrication

## Abstract

Pneumatic artificial muscles (PAMs) are widely used as soft actuators in biomechanics and robotics, yet their adoption in laboratories is often hindered by skill-dependent fabrication processes or reliance on costly commercial products. This article presents an open-source Low-Cost and Tough Pneumatic Artificial Muscle (LT-PAM) designed for research and educational use, with an emphasis on ease of fabrication, reproducibility, and mechanical robustness. The actuator uses a silicone inner tube, a braided sleeve, and mechanically crimped metal fittings that eliminate the need for adhesive bonding in load-bearing connections. All components are assembled from off-the-shelf parts and simple 3D-printed end plugs, enabling rapid and repeatable in-house fabrication. The basic mechanical characteristics are evaluated through quasi-static force–length tests under constant pressure and pressure–contraction tests under constant load, confirming canonical McKibben-type behavior with low sample-to-sample variability. Rupture tests and long-term cyclic tests further demonstrate robustness, with specimens sustaining tensile loads of approximately 600–800 N and surviving more than 9000 pressurization cycles without catastrophic failure. These results indicate that the LT-PAM is a practical, low-cost, and reproducible soft actuator for experiments with high-load soft robots, including musculoskeletal robotic systems.

## Graphical abstract

**Figure:**
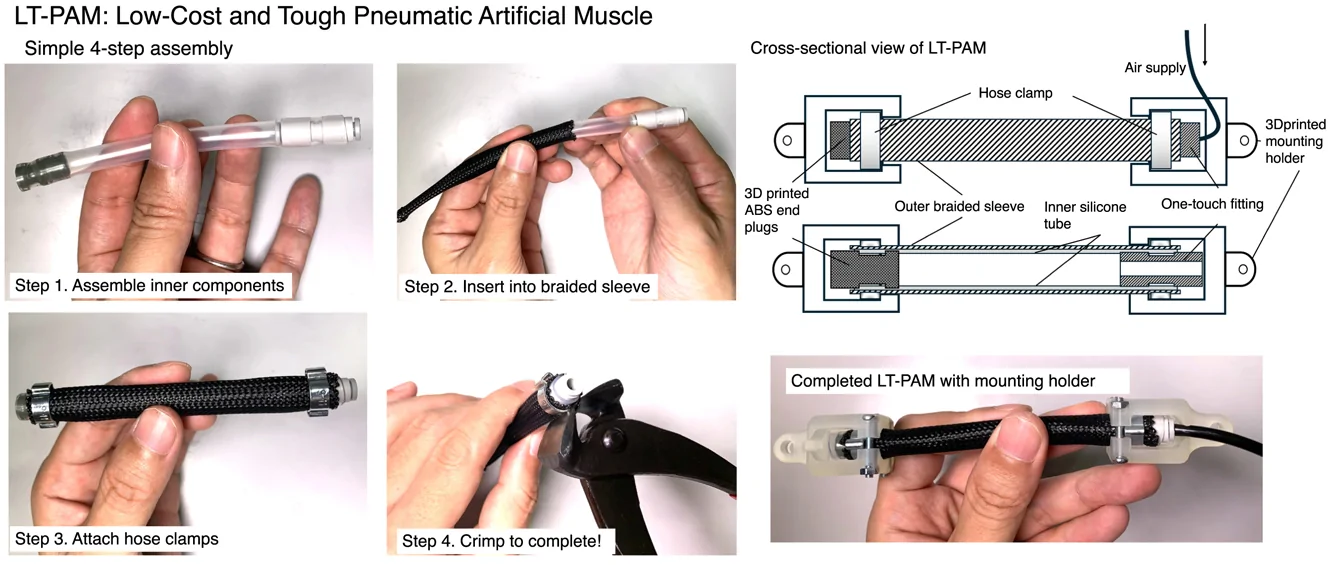

## Specifications table

**Table:** 

**Hardware name**	Low-Cost and Tough Pneumatic Artificial Muscle (LT-PAM)
**Subject area**	Soft robotics, pneumatic actuators
**Hardware type**	Pneumatic artificial muscle actuator
**Closest commercial analog**	Festo Fluidic Muscle DMSP/MAS and s-muscle Co., Ltd. thin McKibben muscles
**Open-source license**	CERN-OHL-P-2.0 for hardware design files (as defined by the Open Source Hardware Association [1]); CC BY 4.0 for documentation
**Cost of hardware**	Approx. USD 5 per 150 mm actuator (about 800 JPY at the time of writing; marginal material cost only, excluding reusable tools; see the note on cost in the Bill of materials section)
**Source file repository**	https://doi.org/10.5281/zenodo.18665922 (Zenodo)

## 1 Hardware in context

Pneumatic artificial muscles (PAMs) have been proposed, developed, and commercialized in various forms as soft actuators that combine flexibility with high power density [2], [3], [4]. Among these devices, McKibben-type artificial muscles are widely used in research and industrial applications because of their relatively simple structure, axial contraction under internal pressure, and mechanical behavior resembling that of biological muscles [5], [6].

In laboratory and educational settings, researchers typically choose between two extremes. At one end are low-cost, handmade McKibben actuators based on community-shared recipes [7], [8], [9] or lab-specific know-how; these actuators often rely on skill-intensive procedures such as wire wrapping or adhesive-based end fixation. Although these approaches offer flexibility and low material cost, they frequently suffer from large sample-to-sample variability in performance and durability. At the other end are proprietary commercial products offered by companies such as Festo [10] and university spin-off companies such as s-muscle Co., Ltd. [11], [12], which provide high performance and reliability but at substantially higher cost and with limited customizability for experimental prototyping.

The Low-Cost and Tough Pneumatic Artificial Muscle (LT-PAM) presented in this work is intended to bridge this gap. It builds directly upon an openly shared McKibben fabrication recipe previously developed by some of the present authors [13], and extends that recipe into a fully dimensioned, openly licensed, and experimentally characterized hardware design. The actuator consists of a silicone inner tube, a braided sleeve, and mechanically crimped metal fittings. Following this approach, external loads are carried primarily by the braid and crimped hardware, while the inner tube functions as a pressure bladder. This configuration avoids reliance on adhesive bonding or curing-dependent processes for load-bearing structural fixation, reduces operator-skill dependence, and suppresses typical failure modes such as end-cap blowout or braid slippage. The protruding geometry of the crimp hardware likewise serves as a simple mechanical hook, enabling rapid attachment to and detachment from robotic structures without specialized tools.

Rather than aiming to outperform all existing PAMs, the LT-PAM design is evaluated against practical criteria derived from typical use cases in musculoskeletal robotics and other high-load soft robotic systems. These criteria include sustaining tensile loads on the order of several hundred newtons, withstanding on the order of $10^{4}$ pressurization cycles without catastrophic failure, and being fabricated reproducibly from off-the-shelf parts and 3D-printed end plugs in a standard university laboratory. The resulting actuator is inexpensive enough for deployment in multi-muscle systems and robust enough for repeated, high-load experiments, such as multi-actuator soft-robot trials or upper-limb assistive-motion studies.

A quantitative comparison further clarifies this positioning. Table 1 compares the LT-PAM with two representative commercial McKibben-type actuators — the Festo Fluidic Muscle DMSP/MAS [10] and the thin McKibben muscles supplied by s-muscle Co., Ltd. [11], [12] — and two openly shared do-it-yourself recipes: the Open Soft Machines mini-McKibben [8] and the Soft Robotics Toolkit actuator [9]. Because the actuators differ in size and test conditions, manufacturer- or publication-specified values are reported directly, and unavailable entries are marked “N/R” rather than assigned qualitative rankings. The comparison shows that the distinctive contribution of the LT-PAM is not a single peak specification but rather a combination of attributes: a recommended working load of about 400 N (with rupture between 600 and 800 N), durability beyond 9000 cycles, a material cost of about USD 5, reproducible in-house fabrication enabled by crimp-based end fixation, and a permissively licensed, fully open design that permits commercial reuse. In particular, the material cost is far below that of commercial units; compared with other do-it-yourself recipes — whose per-unit costs are of a similar order — the advantages of the LT-PAM lie in reproducibility, load capacity, characterized durability, and open licensing rather than in cost alone.

A key appeal of PAMs for musculoskeletal robotics is variable-stiffness actuation. In an antagonistic pair, the joint equilibrium position is set by the difference between the two muscle forces and the joint stiffness by their sum, so co-contraction tunes stiffness independently of position, switching between stiff, robust and soft, compliant behavior [14]. This follows from the monotonic, pressure-dependent force–length curve of each muscle (Section 7.2.1). The low cost and reproducibility of the LT-PAM make it well suited to exploring such behavior across many settings, rather than being optimized for a single task.

In terms of biomechanical scale, a single LT-PAM (recommended working load ∼400 N, rupture 600–800 N) is of the order of individual muscle forces in upper-limb and sub-body-weight assistive tasks. Higher-force regimes, such as lower-limb locomotion under full body weight (kN-scale tendon forces [15]), are anticipated to be addressed by using several actuators in parallel rather than by optimizing a single unit; the low per-unit cost and the compact end fittings are intended to make such bundling practical (see also Section 7.2.4). The LT-PAM is therefore intended as a general, scalable building block rather than an actuator tuned to a single anatomical target.

**Table 1:** Comparison of the LT-PAM with representative commercial and do-it-yourself McKibben-type pneumatic artificial muscles. Manufacturer-specified or publication-reported values are reported directly; “N/R” denotes not reported. ‡ denotes an order-of-magnitude estimate of marginal material cost based on component retail prices, because the do-it-yourself recipes do not publish a per-unit cost.

Attribute	LT-PAM (this work)	Festo DMSP/MAS	s-muscle thin McKibben	Niiyama mini-McKibben	Soft Robotics Toolkit/DIY
Type	Open-source, in-house	Commercial	Commercial	Open recipe (web)	Open recipe (web, research)
Material cost (per unit)	≈**USD 5 (**≈**800 JPY)**	Commercial (quotation-based)	Commercial (quotation-based)	Est. ≈3–5 USD${}^{‡}$	Est. ≈2–6 USD${}^{‡}$
Max. force/load	Rupture 600–800 N; working ≤400 N (SF 1.5)	≈140–6000 N (ϕ5–40)^a^	≈30–280 N (model-dependent)^a^	Low; N/R	Design-dependent; N/R
Contraction ratio	≈25% (no load)	≈25% (20% for ϕ5)	EM ≈20%–24%; KM ≈31%–33%	∼25% (N/R)	∼25% (up to 40%)
Operating pressure	0–0.8 MPa	0–0.6 MPa (ϕ5)	≤0.3 MPa (EM); ≤0.2 MPa (KM)	Low (N/R)	Low (N/R)
End fixation	Metal crimp (no adhesive)	Molded press fittings	User-processed (M3 tap)	Cable ties	Clamp/tie
Durability	>9000 cycles; gradual (slow-puncture) failure	$10^{5}$–$10^{7}$ strokes (mfr., cond.-dep.)	>$10^{6}$ cyc. at 0.3 MPa (EM, ref.)	N/R	N/R
Customizability	Length, braid diameter, and plug freely adjustable	ϕ5–40 mm; length 40–9000 mm	Tube OD 1.3–10 mm (EM/SM/KM)	Adjustable in recipe	Adjustable in recipe
Fabrication/tooling	Easy; no adhesive; low skill dependence; crimp tool (≈2,290 JPY)	Purchase only	Purchase only	Easy (scissors, ties; ∼60 min)	Moderate (molding, braiding)
Recipe open/in-house	**Open; in-house (Zenodo)**	No/no	No/no	Open/yes	Open/yes
License	**CERN-OHL-P-2.0 + CC BY 4.0**	Proprietary	Proprietary	Web (no formal license)	Research/non-commercial (patents pending; CC BY-NC-SA 4.0)

a Festo theoretical force: 140/630/1500/6000 N for ϕ5/10/20/40. s-muscle no-load peak force: ≈32/75 N (EM20/EM40 at 0.5 MPa), ≈63/275 N (KM40/KM100 at 0.4 MPa).

## 2 Hardware description

The Low-Cost and Tough Pneumatic Artificial Muscle (LT-PAM) presented in this work is a customizable soft actuator designed to satisfy the practical performance requirements of research-oriented applications, particularly in musculoskeletal robotics and other high-load soft robotic systems. Conventional McKibben actuators often rely on adhesive bonding to fix end fittings; this approach is sensitive to operator skill and requires curing time. The LT-PAM instead employs mechanically crimped metal fittings. This structural choice avoids reliance on adhesive bonding for structural fixation or load transmission, shortens assembly time, and is expected to improve the reproducibility of the assembly process.

The core structure consists of three elements: (1) a silicone inner tube that functions as a pressure bladder, (2) a braided polyester sleeve that constrains radial expansion and converts internal pressure into axial contraction, and (3) 3D-printed end plugs. At each end, a two-ear metal crimp clamp is applied over the sleeve, tube, and plug, mechanically locking all layers simultaneously without adhesive. Under tensile loading, external forces are borne primarily by the braid and the crimped metal hardware, whereas the inner tube is subjected mainly to internal pressure. This functional separation suppresses common failure modes observed in adhesive-based designs, such as end-cap blowout, delamination, and braid slippage.

The LT-PAM is not intended to compete directly with high-end commercial pneumatic artificial muscles in absolute performance. Instead, its design philosophy is to meet the minimum but sufficient mechanical and durability requirements for laboratory-scale experiments while remaining inexpensive, reproducible, and fully manufacturable within a standard university environment. The required specifications were informed by simple order-of-magnitude estimates based on representative biomechanical loads and typical experimental use cases in musculoskeletal and soft robotics [15], [16], [17], providing a rational basis for actuator sizing without over-specialization.

In addition to load capacity, repeated actuation is a critical consideration in experimental robotics, where motion trials often involve thousands of contraction–extension cycles. The LT-PAM was therefore designed with durability and predictable failure behavior under repeated use in mind. Within the intended load range and during cyclic operation, degradation tends to manifest gradually, for example through slow air leakage or progressive loosening of fittings, rather than by immediate structural rupture. Under excessive or off-design loading conditions, however, rapid failure may still occur, as is typical for soft actuators. This observable and gradual degradation during repeated laboratory use improves safety and risk awareness during long-duration experiments and prototyping.

Another key advantage of the LT-PAM is its manufacturability and scalability. The actuator can be fabricated entirely from off-the-shelf components and a small number of 3D-printed parts using standard desktop tools. Because structural fixation and load transmission do not rely on adhesive bonding, long curing times are unnecessary; as a result, assembly is rapid, material cost is low, and performance is expected to be less dependent on operator expertise. The only adhesive used, an ABS cement, is non-structural: it smooths the layer lines of the ABS 3D-printed plug to improve sealing and does not contribute to mechanical strength. This feature makes the design particularly suitable for laboratories that require multiple identical actuators for comparative studies, multi-muscle robotic systems, or hands-on educational use.

Taken together, the design considerations described above — crimp-based, adhesive-free end fixation, off-the-shelf and 3D-printed components, and a load path that separates structural and pressure-bearing functions — translate into the following practical advantages for research and educational use:

• **Customizability:** Actuator length, braided-sleeve diameter, and end-plug geometry can be readily modified to match specific robotic configurations or experimental constraints.
• **Cost-effectiveness:** The low material cost allows large-scale deployment in multi-actuator systems without prohibitive expense.
• **Reproducibility:** Crimp-based end fixation removes the skill-dependent adhesive step and yielded consistent mechanical behavior across samples prepared by a single fabricator.
• **Safety and reliability:** During repeated use within the intended operating range, degradation tends to occur gradually, which improves safety during prototyping and experimentation.
• **Lab manufacturability:** The design requires no specialized equipment and supports rapid, fully in-house fabrication.

These characteristics make the LT-PAM an accessible, research-grade soft actuator well suited for musculoskeletal robotics [14], [18], other high-load soft robotic systems, and educational prototyping environments.

## 3 Design files

Repository: https://doi.org/10.5281/zenodo.18665922

• **STL files for 3D-printed parts** LT-PAM_stopper.stl, LT-PAM_holder.stl, LT-PAM_longholder.stl
• **CAD source files (Autodesk Inventor .ipt format)** LT-PAM_stopper.ipt, LT-PAM_holder.ipt, LT-PAM_longholder.ipt
• **Demonstration video** LT-PAM_HowToMake.mp4 — Video demonstrating LT-PAM assembly and operation
• **Bill of materials and documentation** BOM_LT-PAM.csv (editable bill of materials), README.md (repository documentation)

**Table:** 

Design filename	File type	Open-source license	File location
LT-PAM_stopper.stl	STL (3D model for 3D printing)	CERN-OHL-P-2.0	https://doi.org/10.5281/zenodo.18665922
LT-PAM_stopper.ipt	CAD source file (Autodesk Inventor)	CERN-OHL-P-2.0	https://doi.org/10.5281/zenodo.18665922
LT-PAM_holder.stl	STL (3D model for 3D printing)	CERN-OHL-P-2.0	https://doi.org/10.5281/zenodo.18665922
LT-PAM_holder.ipt	CAD source file (Autodesk Inventor)	CERN-OHL-P-2.0	https://doi.org/10.5281/zenodo.18665922
LT-PAM_longholder.stl	STL (3D model for 3D printing)	CERN-OHL-P-2.0	https://doi.org/10.5281/zenodo.18665922
LT-PAM_longholder.ipt	CAD source file (Autodesk Inventor)	CERN-OHL-P-2.0	https://doi.org/10.5281/zenodo.18665922
LT-PAM_HowToMake.mp4	Demonstration video (MP4)	CC BY 4.0	https://doi.org/10.5281/zenodo.18665922
BOM_LT-PAM.csv	Editable bill of materials (CSV)	CC BY 4.0	https://doi.org/10.5281/zenodo.18665922
README.md	Repository documentation (Markdown)	CC BY 4.0	https://doi.org/10.5281/zenodo.18665922

**LT-PAM_stopper.stl** — Printable STL model of the ABS end plug used to seal the silicone inner tube and interface with the pneumatic fitting.

**LT-PAM_stopper.ipt** — Editable CAD source (Autodesk Inventor) of the end-plug geometry, intended for modifying the tube diameter or other design parameters.

**LT-PAM_holder.stl** — Printable STL model of the LT-PAM mounting bracket. The bracket hooks onto the protruding crimp geometry of the end fitting, allowing the actuator to be rapidly attached to or detached from a robot or experimental apparatus.

**LT-PAM_holder.ipt** — Editable CAD source (Autodesk Inventor) of the mounting bracket, intended for adapting the hook geometry or mounting interface to a specific robot structure.

**LT-PAM_longholder.stl** — Printable STL model of the extended mounting bracket, providing greater structural reach than LT-PAM_holder.stl for applications where additional clearance is required.

**LT-PAM_longholder.ipt** — Editable CAD source (Autodesk Inventor) of the extended mounting bracket.

**LT-PAM_HowToMake.mp4** — A short instructional video demonstrating the assembly process and basic operation of the LT-PAM for laboratory and educational use.

**BOM_LT-PAM.csv** — Editable bill of materials listing every off-the-shelf component with its supplier, part number, quantity, and unit cost.

**README.md** — Repository documentation describing each file, the recommended print and assembly settings, the license, and the citation, so that the design can be reproduced and reused outside the originating laboratory.

**Repository contents and relation to the fabrication workflow.** The repository contains all files required to reproduce the LT-PAM: (i) STL files for the 3D-printed parts (the end plug and the mounting holders) used in the build instructions; (ii) the editable Autodesk Inventor (.ipt) source files for these parts, which provide the complete part geometry and dimensions and allow the tube diameter, plug geometry, and hook geometry to be modified; (iii) an editable bill-of-materials file listing every off-the-shelf component with its supplier and part number; and (iv) an assembly and operation demonstration video. The 3D-printed parts do not require a printer-specific profile; standard fused deposition modeling (FDM) settings with 100% infill are sufficient to ensure airtightness of the end plug. Therefore, only these key parameters are specified, rather than a full slicer profile. All files use descriptive, self-explanatory names and are documented in a README that describes each file together with the recommended print and assembly settings, providing sufficient supporting information for readers outside the originating laboratory to reproduce, reuse, and modify the design.

**Fig. 1:**
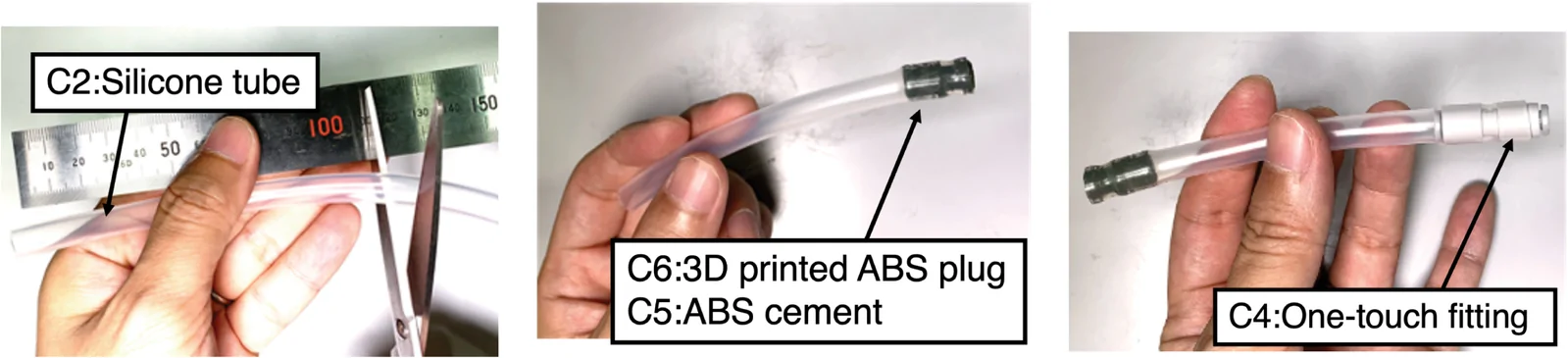
Step 1 — Tube-side assembly (C2, C6, C4): (left) cutting the silicone tube (C2) to the required length; (center) inserting the ABS end plug (C6) into the tube; (right) attaching the pneumatic straight union fitting (C4).

**Fig. 2:**
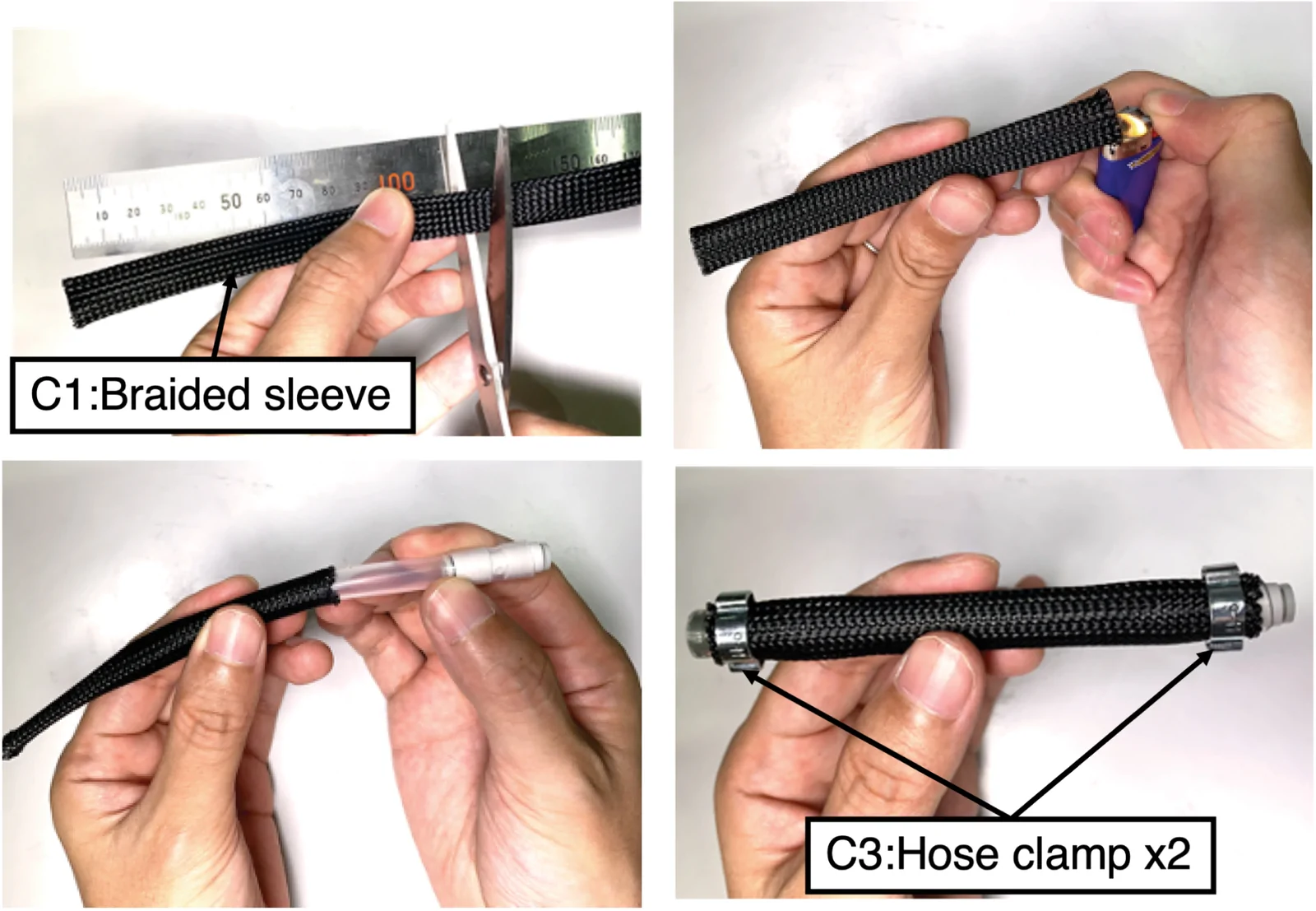
Step 2 — Sleeve integration (C1, C3): (top-left) cutting the braided sleeve (C1) to length; (top-right) heat-sealing the cut ends with a lighter to prevent fraying; (bottom-left) inserting the Step-1 assembly into the sleeve; (bottom-right) positioning the two-ear crimp clamps (C3) at both ends.

## 4 Bill of materials

This project uses a pneumatic artificial muscle (PAM) based on the McKibben structure and optimized for laboratory and educational use. A complete, editable bill of materials (BOM) is available in the Zenodo repository: https://doi.org/10.5281/zenodo.18665922

The actuator is designed for 4 mm-outer-diameter pneumatic tubing and consists of an inner silicone tube enclosed in a braided sleeve. End fittings and sealing caps are fabricated by 3D printing. A small number of tools are required for assembly.

**Pneumatic artificial muscle materials:**

• Denka Electron NFL-9, fray-resistant braided sleeve, ¥648/m
• Monotaro MGJG-8 × 10, silicone tube (ID 8 mm, OD 10 mm), ¥279/m
• ESCO EA463AE-11, hose clamp (9–11 mm, two-ear type), ¥506/10 pcs (¥50.6 each; 2 pcs per actuator)
• SMC KQ2H04-00 A, one-touch straight union fitting (metric, 4 mm), ¥159 each (2 pcs per actuator)
• Tamiya ITEM 87137, ABS cement, ¥278/bottle (non-structural; smooths the layer lines of the ABS 3D-printed plugs to improve sealing)
• 3D-printed ABS end plugs (LT-PAM_stopper; 100% infill) — design file available on Zenodo: https://doi.org/10.5281/zenodo.18665922
• 3D-printed mounting holders (LT-PAM_holder or LT-PAM_longholder; compatible with any rigid printable material such as PLA, ABS, or UV-curable resin; FDM or resin printer) — connect the actuator to the robotic structure or test rig; design file available on Zenodo: https://doi.org/10.5281/zenodo.18665922

**Assembly tool:**

• Apollo crimping tool, ¥2,290

**Table:** 

Designator	Component	Number	Unit cost (JPY)	Total cost (JPY)	Material source	Material type
C1	Braided sleeve (NFL-9)	1 m	648	648	Denka Electron	Polymer
C2	Silicone tube (MGJG-8 × 10)	1 m	279	279	Monotaro	Polymer
C3	Hose clamp (EA463AE-11)	2 pcs	50.6	101.2	ESCO	Metal
C4	One-touch fitting (KQ2H04-00A)	2 pcs	159	318	SMC	Metal
C5	ABS cement (ITEM 87137)	1	278	278	Tamiya	Other
C6	3D printed ABS end plugs (LT-PAM_stopper)	2 pcs	–	–	Zenodo/internal	Polymer
C7	3D-printed mounting holders (LT-PAM_holder or LT-PAM_longholder; any rigid material)	2 pcs	–	–	Zenodo/internal	Polymer
T1	Crimping tool	1	2290	2290	Apollo	Metal

*Note on cost:* The prices above are listed per purchase unit (for example, per 1 m of silicone tube or braided sleeve, and per 10-piece pack of hose clamps), so their sum is not the cost of a single actuator. The per-actuator figure quoted in the Specifications table (approximately USD 5, about 800 JPY at the time of writing) is the marginal material cost of building one 150 mm actuator: roughly 0.2 m each of silicone tube (C2) and braided sleeve (C1), two hose clamps (C3), and two one-touch fittings (C4). The two fittings and two clamps (≈420 JPY) dominate this cost. The reusable crimping tool (T1) is excluded, and the 3D-printed parts (C6, C7) and the ABS cement used only for surface finishing (C5) contribute a negligible per-actuator cost.

**Fig. 3:**
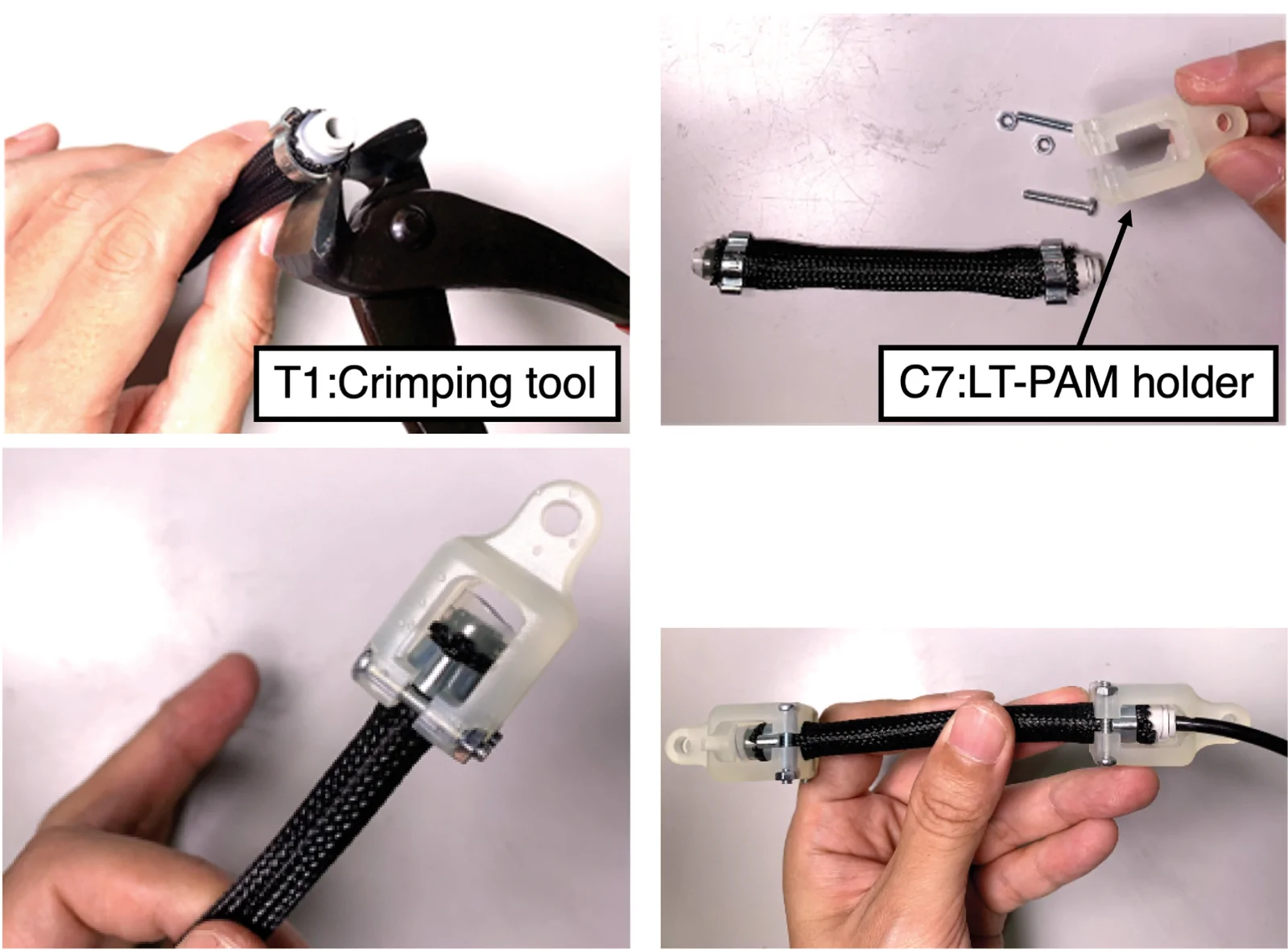
Step 3 — End-fixation and completed actuator (C3, T1): (top-left, top-right) crimping the two-ear clamps (C3) with the Apollo tool (T1); (bottom-left) attaching the external connector module and securing with M3 screws; (bottom-right) the completed LT-PAM ready for use.

## 5 Build instructions

This section provides a fully reproducible, step-by-step fabrication procedure for the Low-Cost and Tough Pneumatic Artificial Muscle (LT-PAM). All component designators (C1–C7, T1) follow the **Bill of Materials summary**. Photographs illustrating the major steps are provided in Fig. 1, Fig. 2, Fig. 3.

The LT-PAM consists of the following primary components:

• Silicone inner tube (C2)
• Braided sleeve (C1)
• 3D-printed end plugs (C6)
• Pneumatic straight union fitting (C4)
• Two-ear metal crimp clamps (C3)
• 3D-printed mounting holder (C7; required for robotic integration or tensile testing)

Crimping is performed with the Apollo tool (T1). No adhesive is required for structural fixation; the ABS cement (C5) is optional and non-structural, used only to smooth the layer lines of the ABS 3D-printed plug and thereby improve its airtight seal.

### 5.1 Step 1: Preparation of the tube-side assembly (C2, C6, C4)

#### Step 1-1: Cutting the silicone tube (C2).

Cut the MGJG-8 × 10 silicone tube to the required length. The cutting operation is illustrated in Fig. 1 (left). Ensure that the cut is perpendicular to the tube axis to achieve airtight sealing.

#### Step 1-2: Inserting the end plug (C6).

Apply a thin coat of ABS cement (C5; optional, non-structural) to the ABS 3D-printed end plug to smooth its layer lines and improve the airtight seal. Insert the plug fully into the tube until it is seated. This corresponds to Fig. 1 (center).

#### Step 1-3: Installing the straight pneumatic union (C4).

Fit the SMC straight union (KQ2H04-00 A) into the plug as shown in Fig. 1 (right). This completes the pressure-inlet assembly.

### 5.2 Step 2: Assembly of tube and braided sleeve (C1, C3)

#### Step 2-1: Cutting the braided sleeve (C1).

Cut the NFL-9 sleeve so that it is slightly longer than the silicone tube. This step is illustrated in Fig. 2 (top-left). The NFL-9 sleeve was selected for its fray resistance and dimensional compatibility with the silicone tube used in this design; sleeves of different diameters or materials may be substituted to adjust contraction ratio or force output, provided that the inner diameter matches the outer diameter of the silicone tube.

#### Step 2-2: Heat-sealing the sleeve ends.

Gently melt the cut ends with a lighter to prevent fraying. This makes assembly smoother and maintains structural integrity. See also Fig. 2 (top-right).

#### Step 2-3: Sliding the step-1 assembly into the sleeve.

Insert the entire tube-plug-union assembly into the braided sleeve. This corresponds to Fig. 2 (bottom-left). Ensure that the sleeve fully covers both plug regions.

#### Step 2-4: Positioning the crimp clamps (C3).

Place a two-ear clamp at each end so that it overlaps the underlying plug or fitting, as shown in Fig. 2 (bottom-right). Proper overlap ensures that axial forces are transmitted through the clamp and braid.

### 5.3 Step 3: End-fixation and connector-mounting (C3, T1)

#### Step 3-1: Crimping the clamps (C3).

Using the Apollo crimping tool (T1), compress both ears of each clamp until the tube, sleeve, and plug form a single integrated body. The crimping motion is depicted in Fig. 3 (top-left and top-right). **At this point, the LT-PAM itself (the actuator body) is complete.**

#### Step 3-2: Attaching the mounting holder (C7).

Fit the 3D-printed mounting holder (C7; LT-PAM_holder or LT-PAM_longholder) over the end hardware to prepare it for robotic mounting or tensile testing. The protruding crimp geometry acts as a mechanical hook, enabling rapid attachment and detachment using only standard hand tools. See Fig. 3 (bottom-left).

#### Step 3-3: Securing with M3 screws.

Fix the mounting holder (C7) using M3 screws. This step completes the mechanical integration of the actuator into an experimental rig.

After assembly, perform the leak test described in Section 6 (Operation instructions) before using the actuator.

## 6 Operation instructions

This section describes proper use of the LT-PAM in a typical laboratory environment. These procedures apply to general experimental use; specific characterization protocols, such as those in Section 7, should be implemented as extensions of this baseline.

### 6.1 Initial setup and leak testing

Before first use and after assembly, verify that the LT-PAM is leak-free:

1. Mount the LT-PAM on the mechanical fixture using the mounting holders (C7). Ensure proper axial alignment with the intended direction of motion.
2. Connect pneumatic tubing (4 mm OD) to the one-touch fitting (C4). Attach the supply line to a regulated compressed-air source with a pressure gauge.
3. **Leak test:** Gradually pressurize the actuator to 0.2–0.3 MPa. Submerge the LT-PAM in water and visually inspect for bubbles. If leakage is detected, depressurize immediately and either re-crimp C3 or replace the plug (C6).
4. Once leak-free operation is confirmed, the LT-PAM is ready for experimental use.

### 6.2 Safety guidelines

**Always observe the following safety precautions during operation:**

• **Pressure limit:** Do not exceed 0.8 MPa internal pressure. Higher pressures may cause rupture of the silicone tube (C2) or failure of 3D-printed components (C6).
• **Avoid twisting or sharp bending:** Operating the actuator in severely bent or twisted configurations can induce non-uniform stress and premature failure.
• **Protective equipment:** Wear safety glasses during all pressurized operations. In the event of sudden failure, the actuator may exhibit a whipping motion.
• **Secure mounting:** Ensure that the LT-PAM is firmly attached to the test rig or robotic fixture to prevent unintended movement.
• **Depressurize before handling:** Always vent the actuator to atmospheric pressure before adjusting fittings, repositioning, or replacing components.
• **Pre-use inspection:** Before each experiment, inspect the braided sleeve (C1) for fraying, the crimp clamps (C3) for loosening, the end plugs (C6) for cracks, and the fittings (C4) for looseness; then briefly pressurize the actuator and listen for air leakage. Do not use an actuator showing any of these signs until the affected part is replaced.

**Component replacement and failure cues.** The LT-PAM has no fixed replacement interval; instead, audible air leakage provides a practical maintenance cue. In our experience, degradation of the silicone tube, end plugs, or fittings usually produces a hissing sound caused by leakage before any rupture or structural failure, so replacing the component responsible for the leakage is recommended as a precaution. The recommended working-load limit of about 400 N — a safety factor of 1.5 below the 600–800 N rupture range — is based on rupture behavior. Even below this limit, fatigue- or wear-induced leakage can still develop over prolonged use; such degradation is gradual and is typically preceded by audible leakage, so we recommend sound-triggered inspection and replacement rather than a fixed cycle count.

### 6.3 Standard operating procedure

For typical experimental use, follow these steps:

1. **Pre-operation check:** Confirm that all pneumatic and mechanical connections are secure. Verify that the pressure regulator is set to the desired operating range (typically 0.0–0.8 MPa).
2. **Gradual pressurization:** Increase internal pressure slowly (recommended ramp rate: ≤0.1 MPa/s) to avoid dynamic overshoot or mechanical shock. Monitor actuator contraction and generated tension as required by the experiment.
3. **Data acquisition:** Record relevant quantities such as displacement, force, and internal pressure using appropriate sensors and data acquisition systems.
4. **Pressure cycling (if applicable):** For repeated actuation trials, allow sufficient time between cycles for the actuator to return to its initial state if necessary.
5. **Depressurization:** After completing each trial, reduce the pressure to atmospheric pressure before changing load conditions, repositioning the actuator, or ending the session.

### 6.4 Post-experiment inspection and storage

At the conclusion of an experimental session:

• Close the supply valve and fully vent the pneumatic line to atmospheric pressure.
• Visually inspect the braided sleeve (C1) and crimp clamps (C3) for signs of wear, fraying, or loosening. Check the end plugs (C6) for cracks or deformation.
• If any damage is observed, replace the affected components before further use.

### 6.5 Troubleshooting common issues

• **Air leakage detected:** Re-crimp the clamps (C3) using the Apollo tool (T1), or replace the end plug (C6) if damaged. Ensure that the silicone tube (C2) is fully seated on the plug.
• **Reduced contraction performance:** Check for partial obstruction in the pneumatic fitting (C4) or supply line. Verify that the braided sleeve (C1) has not slipped relative to the end fittings.
• **Visible wear or fraying of the braid:** Replace the braided sleeve (C1) and reassemble the actuator following the build instructions in Section 5.

## 7 Validation and characterization

### 7.1 Experimental setups

To ensure reproducible mechanical characterization of the LT-PAM, two complementary experimental rigs were used. These rigs correspond directly to the experiments described in this section:

• Constant-pressure forced-extension tests (Basic experiment 1 and Highlight experiment 1)
• Constant-load pressurization tests (Basic experiment 2 and Highlight experiment 2)

Both rigs were designed to minimize friction, maintain accurate axial alignment, and provide high-resolution measurements of displacement and tension.

#### 7.1.1 Horizontal forced-extension test rig (constant-pressure tests)

**Fig. 4:**
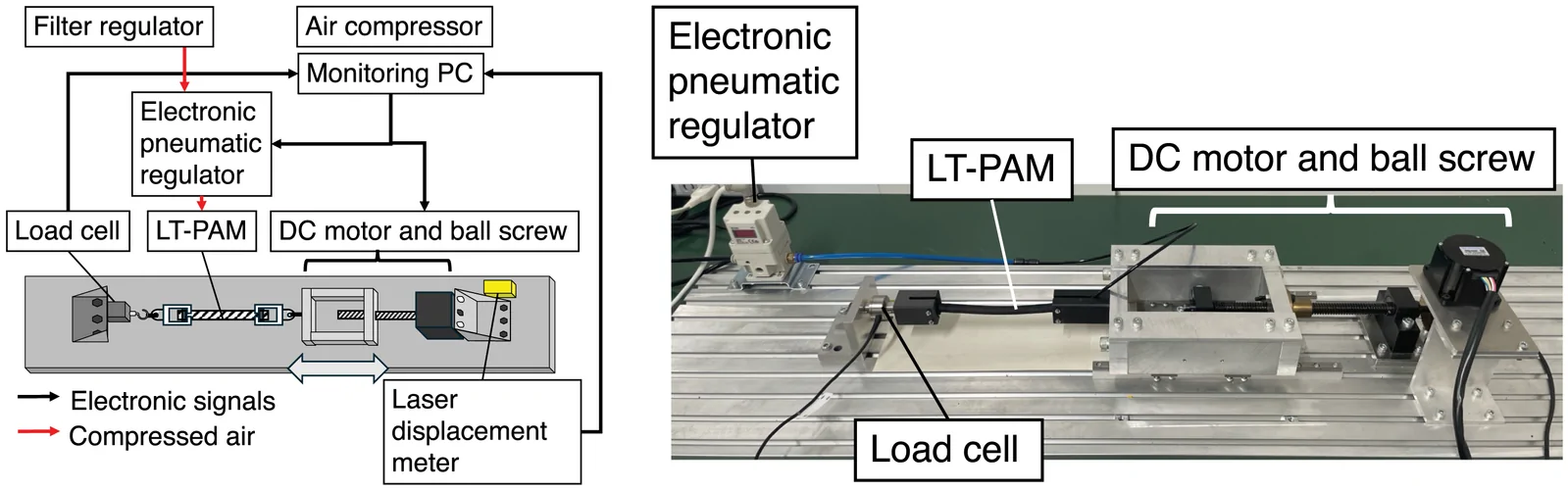
Horizontal forced-extension test rig for constant-pressure characterization. (left) System overview diagram showing the control and measurement architecture. (right) Photograph of the assembled rig; the LT-PAM is mounted between a fixed load cell and a DC motor-driven ball screw stage, and an electronic pneumatic regulator maintains constant internal pressure.

A custom horizontal test apparatus (Fig. 4) was built to characterize force–length behavior under constant internal pressure. The setup consists of:

• A DC motor-driven ball screw stage for controlled extension and contraction of the LT-PAM
• A load cell (1 kN capacity) mounted in series with the LT-PAM
• A laser displacement meter for measuring actuator displacement
• A low-friction linear guide for axial alignment
• An electronic pneumatic regulator (0.0–0.8 MPa) supplied via a filter regulator and air compressor
• Safety shielding for operator protection

During testing, the actuator was pressurized to a fixed pressure, and the ball screw stage imposed controlled extension and contraction. Tension and displacement were recorded simultaneously by the load cell and laser displacement meter, enabling clean measurement of force as a function of length.

#### 7.1.2 Vertical constant-load pressurization rig (pressure–contraction tests)

**Fig. 5:**
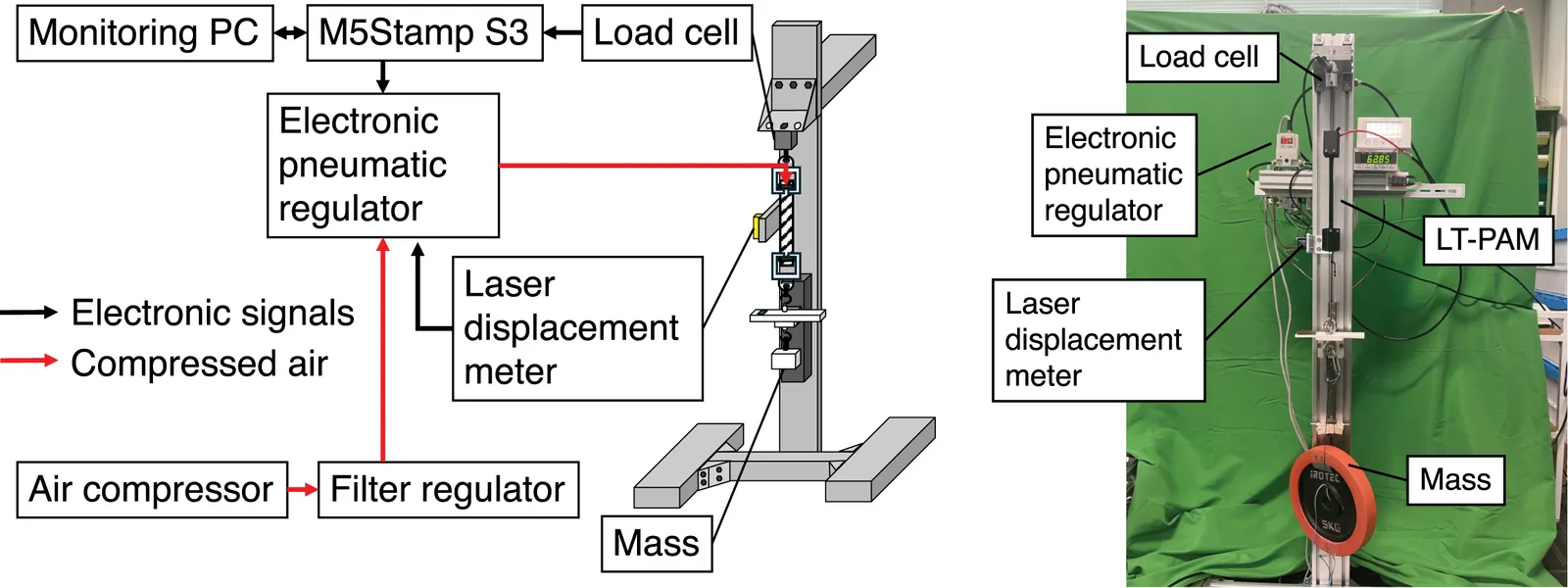
Vertical constant-load pressurization rig for pressure–contraction characterization. (left) System overview diagram. (right) Photograph of the assembled rig; the LT-PAM is suspended vertically between a load cell at the top and a dead-weight plate at the bottom, with actuator length recorded continuously by a laser displacement meter.

A custom vertical test rig (Fig. 5) was used to evaluate contraction behavior under externally applied loads. The system includes:

• A vertical frame with low-friction linear guide rails
• A dead-weight plate (0–300 N) hung from the lower end of the LT-PAM
• A load cell for monitoring tensile force
• A laser displacement meter (resolution 0.01 mm) for measuring actuator length
• An electronic pneumatic regulator (0.0–0.8 MPa) controlled via an M5Stamp S3 microcontroller for periodic actuation
• A filter regulator in the upstream air supply line
• A rigid top connector ensuring axial alignment

This apparatus enables precise measurement of contraction ratio as a function of pressure by applying periodic pressure while maintaining a fixed load.

### 7.2 Results

The mechanical performance of the LT-PAM was evaluated through two complementary sets of experiments. The first set, basic characterization, examined fundamental mechanical behavior under constant pressure and constant load, reported below as the length–tension and pressure–contraction results. The second set, the highlight experiments, emphasized reproducibility, robustness, and structural limits, including sample-to-sample variability evaluated with mean–standard-deviation envelopes and the rupture-strength and repeated-cycle results. All results reported below confirm that the LT-PAM satisfies the key requirements for musculoskeletal and soft robotics research.

#### 7.2.1 Length–tension characteristics under constant pressure

To verify whether the LT-PAM exhibits canonical McKibben-type force–length behavior, quasi-static stretch–release experiments were performed using a horizontal testing apparatus. Each sample was pressurized to one of three levels: 0.3, 0.6, and 0.8 MPa. Five samples were prepared for each pressure level, yielding a total of 15 trials.

In this study, the actuator length L was defined as the length of the contractile section between the inner sides of the two end fittings. In all tests, the initial muscle length was set to $L_{0}=150$  mm, and this value was used as the reference length for strain and contraction calculations. The initial clamp spacing was adjusted to match the initial actuator length.

While pressurized, the actuator was first driven in the contraction direction until the tensile force approached 0–10 N, and was then reversed in the extension direction up to approximately ＋10 mm beyond the initial length. The driving speed was set sufficiently low (∼0.1  mm/s) to ensure quasi-static conditions. Force and displacement were recorded continuously.

**Fig. 6:**
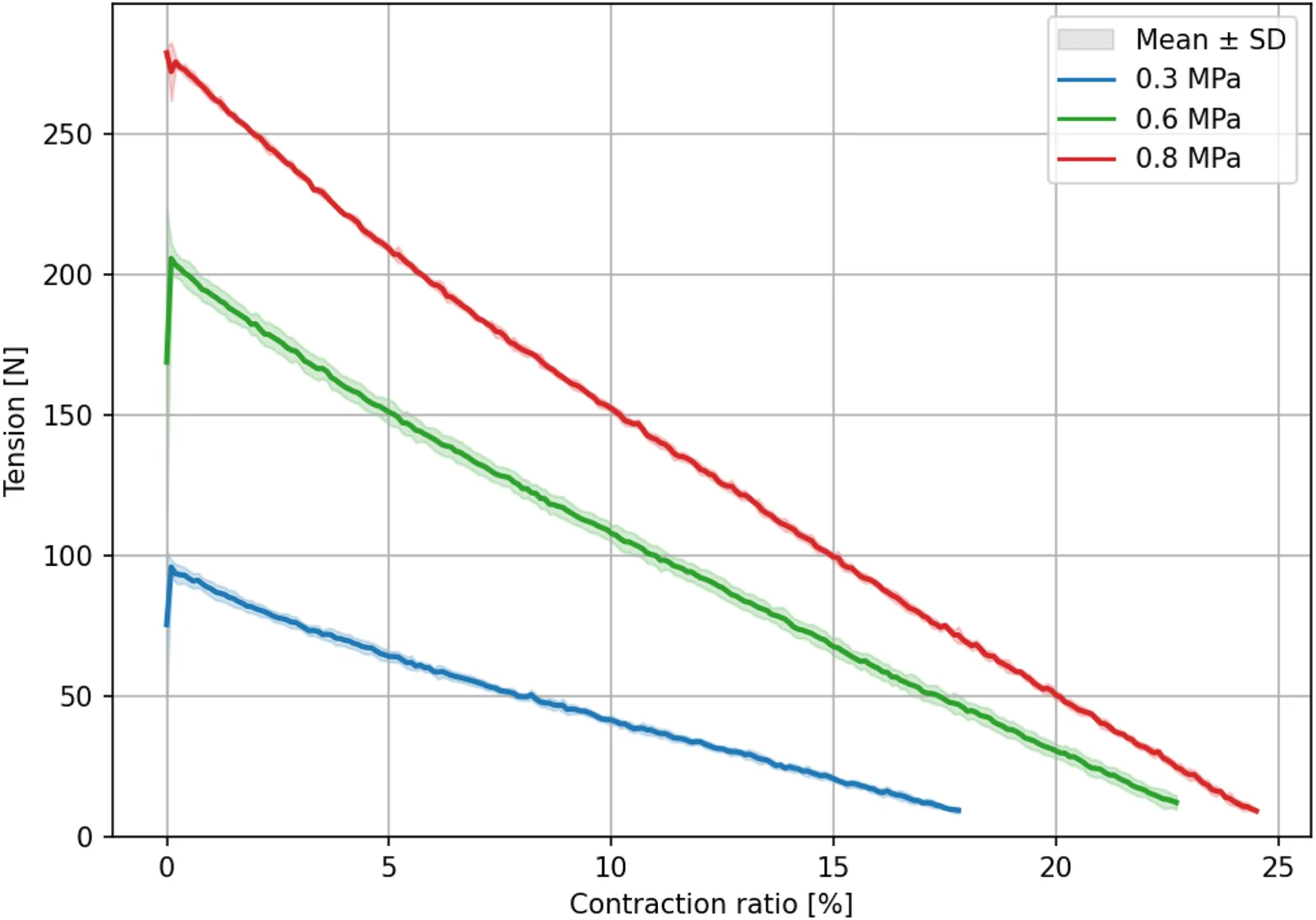
Tension as a function of contraction ratio in the contraction region ($L<L_{0}$) during the length–tension test at three pressure levels (0.3, 0.6, and 0.8 MPa). Within this region, the tension decreases as the contraction ratio increases (equivalently, it increases with actuator length). Solid curves indicate the sample mean; shaded regions indicate ±SD across five samples.

Fig. 6 presents the averaged tension curves in the contraction region ($L<L_{0}$) with shaded regions denoting sample standard deviation. For each pressure, the tension increased with actuator length: within this contraction region ($L<L_{0}$) it decreased monotonically as the contraction ratio increased, while higher internal pressures produced larger tensions. This monotonic force–length trend agrees with the well-established behavior of McKibben-type actuators. The relatively narrow standard-deviation envelopes indicate good sample-to-sample reproducibility within this single-fabricator dataset.

**Fig. 7:**
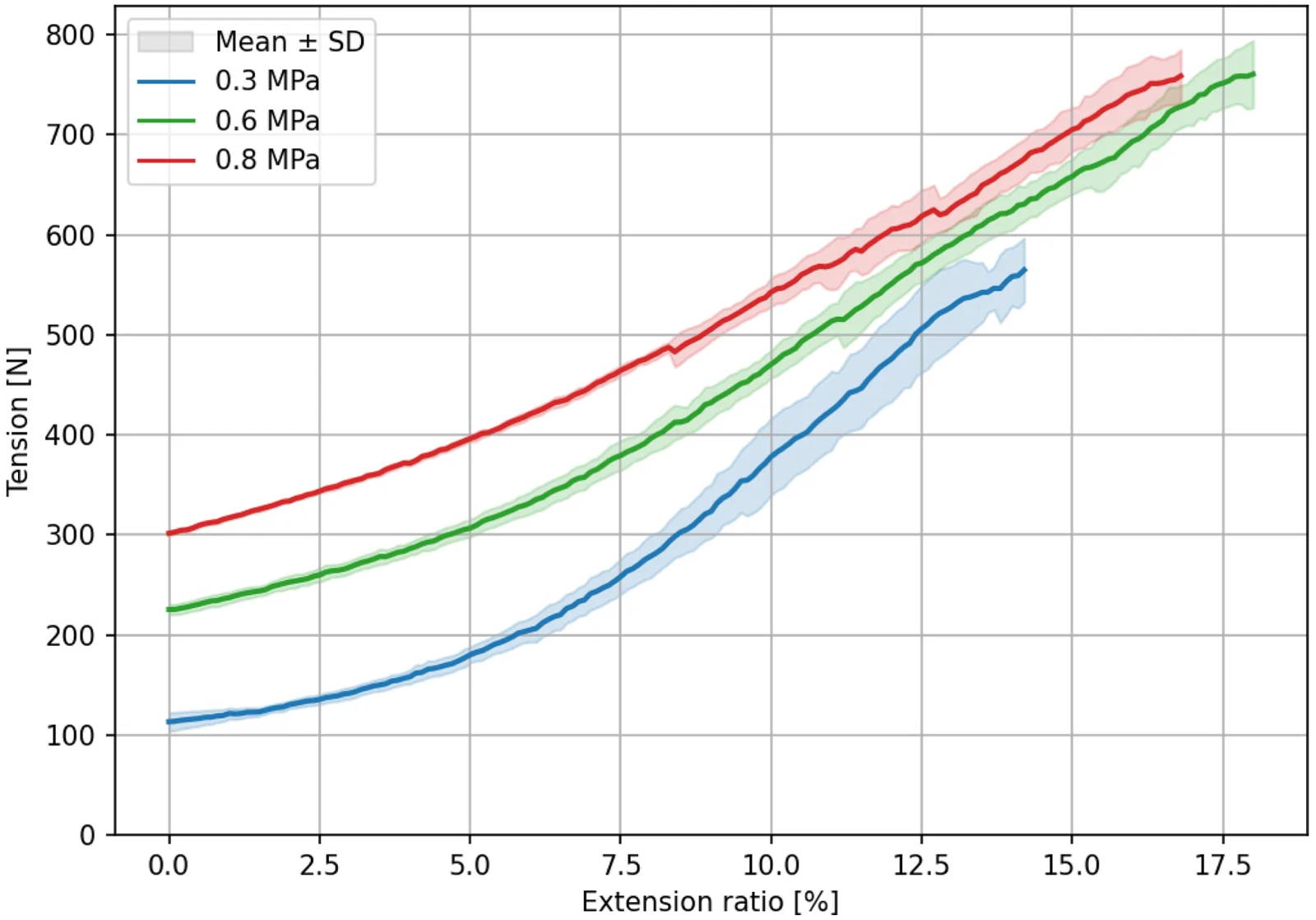
Tension as a function of extension ratio in the extension region ($L>L_{0}$) extracted from the same loading cycle as Fig. 6 at three pressure levels (0.3, 0.6, and 0.8 MPa). Tension increases with the extension ratio, i.e., with actuator length. Solid curves indicate the sample mean; shaded regions indicate ±SD across five samples.

The extension region ($L>L_{0}$) of the same loading cycle is summarized in Fig. 7, where the tension continued to increase with the extension ratio (again rising with internal pressure), consistent with the monotonic increase of tension with actuator length across both regions. These results demonstrate smooth and stable behavior during both contraction and extension without sudden discontinuities, confirming adequate structural robustness under internal pressure.

At low tension, a small onset pressure is required before stable axial contraction develops.

#### 7.2.2 Pressure–contraction characteristics under constant load

To evaluate pressure-dependent contraction behavior under external load, vertical tests were conducted using the custom vertical test rig described above (Section 7, Experimental setups). Four load levels were tested: 0 kg, 10 kg (100 N), 20 kg (200 N), and 30 kg (300 N). Five samples were tested for each condition (20 trials in total). For each trial, pressure was varied between 0.0 and 0.8 MPa in a quasi-static triangular waveform while the actuator length was recorded.

Contraction ratio was computed as

(1) $$ɛ=\frac{L_{0}-L}{L_{0}},$$

where ɛ is the contraction ratio (dimensionless), L is the current actuator length (mm), and $L_{0}=150$ mm is the initial actuator length in the unloaded, unpressurized state, so that ɛ expresses the *net* change in length relative to this reference and therefore combines two superimposed effects: the passive elongation produced by the external dead load and the active contraction produced by the internal pressure.

**Fig. 8:**
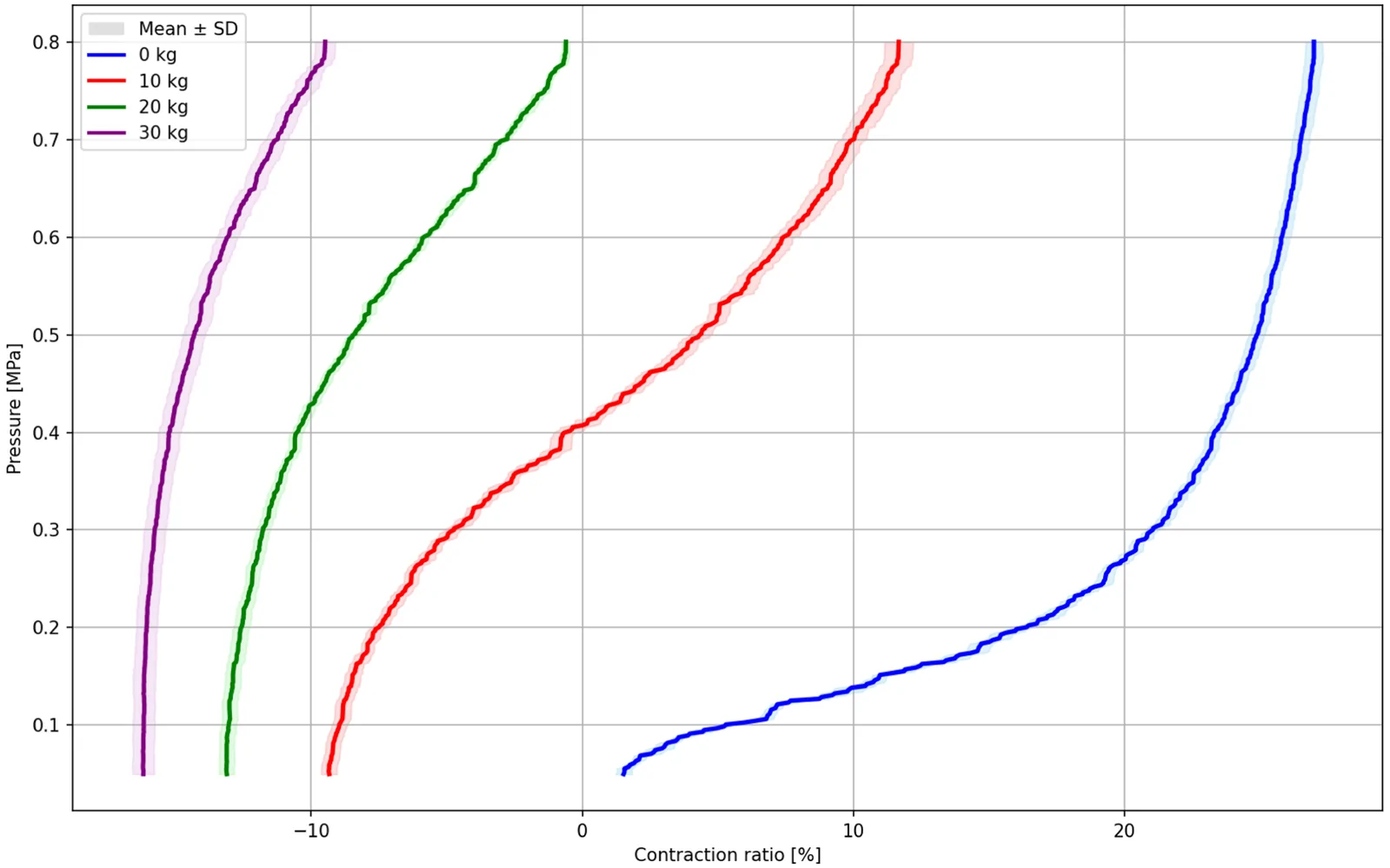
Pressure–contraction characteristics under four load conditions (0–30 kg). Solid curves represent sample means; shaded regions represent ±SD across five samples.

Fig. 8 summarizes the mean ± SD pressure–contraction relationships. Under no load, the LT-PAM reached a maximum contraction of approximately 25%.

The maximum contraction of the LT-PAM can be understood from the classical kinematics of McKibben actuators [5]. Defining the braid angle θ as the angle between an individual sleeve fiber and the actuator’s long axis, fiber inextensibility relates the actuator length to θ, and the enclosed volume is maximized at the neutral angle $\theta_{c}=\arctan\sqrt{2}\approx 54.7{}^{\circ}$, where the generated axial force also vanishes. The free (unloaded) contraction is therefore bounded by

(2) $${ɛ}_{\max}=1-\frac{\cos\theta_{c}}{\cos\theta_{0}},$$

where ${ɛ}_{\max}$ is the maximum unloaded contraction ratio (dimensionless), $\theta_{c}$ is the neutral braid angle (deg), and $\theta_{0}$ is the initial fiber angle (deg). For the braided sleeve used here, the measured initial fiber angle in the unloaded, unpressurized state is $\theta_{0}\approx 32{}^{\circ}$, giving a theoretical contraction ceiling of ${ɛ}_{\max}\approx 32\text{\%}$. The measured no-load contraction of approximately 25% lies slightly below this kinematic ceiling: the difference is governed not by the braid geometry but by the finite dimensions of the internal silicone bladder, whose wall thickness and volume cause it to bunch and self-contact before the braid reaches the neutral angle, so that contraction effectively saturates near θ≈50°.

Although internal pressure always produces active contraction, the superimposed dead load extends the actuator, and under the heavier loads (200 and 300 N) the current length L remains greater than the unloaded natural length $L_{0}$ throughout the pressure range; the active pneumatic contraction is thus outweighed by the load-induced extension, so the net strain ɛ takes negative values. This negative net strain therefore reflects load-induced passive elongation that exceeds the active pneumatic contraction relative to the unloaded reference $L_{0}$, rather than any physical anomaly or structural inconsistency; such load-dependent behavior is characteristic of McKibben-type actuators [4], [5]. If the contraction ratio were instead referenced to the loaded, low-pressure length at each load, the same measurements would describe the active, pressure-induced shortening under that load; here we intentionally use the unloaded natural length as a common reference so that the reported strain captures both the passive load-induced elongation and the active contraction.

All pressure–contraction measurements were obtained under quasi-static conditions, so rate-dependent viscoelastic effects are minimal; any energy dissipation in a full loading–unloading cycle would therefore be expected to be dominated by inter-filament Coulomb friction in the braided sleeve rather than by viscoelasticity of the silicone tube.

#### 7.2.3 Sample-to-sample reproducibility

The standardized crimp fittings, consistent tube geometry, and crimp-based assembly process are intended to keep sample-to-sample variability low. This effect is evident in Fig. 6, Fig. 7, Fig. 8, where the standard-deviation bands remain narrow across all pressure and load conditions. For both contraction and extension phases, SD values typically remained within a few percent of the corresponding mean tension or contraction ratio, indicating uniform mechanical behavior across the tested samples. Because all specimens were fabricated by a single operator, this demonstrates sample-to-sample reproducibility within a single-fabricator dataset; variability across different operators was not assessed and is left for future validation.

#### 7.2.4 Rupture strength

Rupture-strength tests were conducted by extending each specimen under a constant internal pressure of 0.6 MPa until structural failure occurred. Across all samples (n=5), the LT-PAM exhibited failure loads ranging from approximately 600 to 800 N. Some specimens began to show leakage or clamp migration slightly above 600 N, whereas others tolerated loads approaching or exceeding 800 N before degradation. Importantly, no specimen failed by sudden bursting; instead, all specimens degraded gradually through slow leakage, sleeve thinning, or progressive clamp slippage. Under the tested axial loading conditions, the observed degradation modes did not include crushing of the 3D-printed internal plug or rupture initiating at the crimped transition zone, indicating that the crimp interface was not the limiting failure site.

This gradual failure mode is advantageous for safe laboratory operation because it reduces the likelihood of whip-like motion under the tested conditions and provides clear visual and acoustic cues before full rupture. On the basis of these results, and with a conservative safety factor of 1.5, the recommended working-load limit of a single LT-PAM is approximately 400 N for the tested configuration (axial loading of the actuator body). Because the mounting holders, screws, print orientation, and operation near the 0.8 MPa pressure limit were not separately characterized, this recommendation should be re-verified for configurations that differ substantially from the one tested. A parallel bundle of ten actuators would nominally provide a total working-load capacity of roughly 4.0 kN, provided that load sharing among actuators and fixture strength are properly ensured; this is comparable to reported peak Achilles tendon forces in inverse-dynamics studies. We note, however, that bundled operation was not tested in this study. This capacity indicates that the LT-PAM satisfies the tensile-performance requirements of high-load soft-robot and musculoskeletal-robot experiments.

#### 7.2.5 Repeated-cycle robustness under constant load

To evaluate long-term stability under realistic loading conditions, we conducted repeated actuation tests in which each LT-PAM specimen (n=5) was suspended vertically with a constant 200 N load. A square-wave pressure input (0.0–0.6 MPa, period 4 s) was applied for more than 9000 cycles while tension, pressure, and actuator displacement were continuously recorded.

For each cycle, steady-state displacements were extracted from the final 30% of both the low-pressure and high-pressure phases. These values correspond respectively to the maximum extension and maximum contraction states under load. The contraction stroke was computed as the difference between the two steady-state displacements (low minus high), and the contraction ratio was defined relative to the nominal actuator length ($L_{0}=150$  mm).

**Fig. 9:**
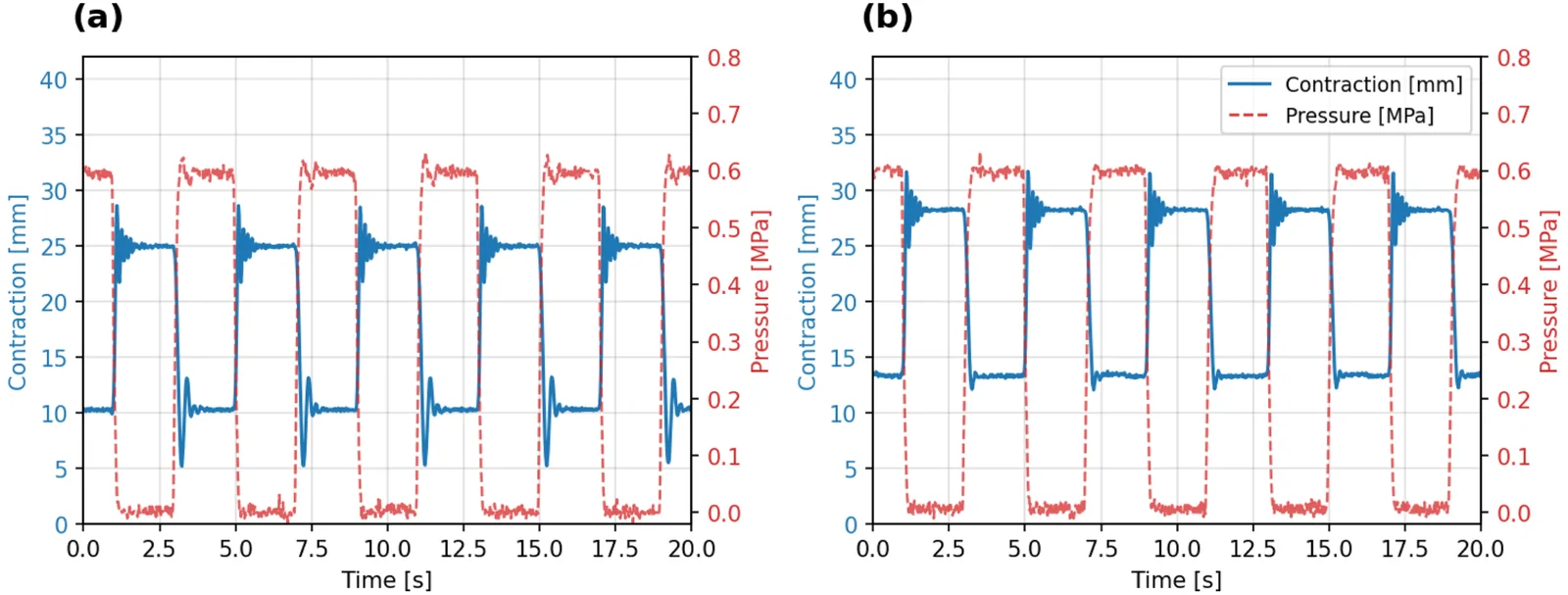
Representative time-domain waveforms of contraction and pressure for a single LT-PAM specimen under a constant 200 N load: (a) early cycles (cycles 101–105) and (b) late cycles (cycles 9001–9005). The waveform shape and contraction amplitude remain highly consistent over more than 9000 cycles, confirming stable long-term actuation performance.

Table 2 summarizes the evolution of high-pressure displacement, low-pressure displacement, and contraction stroke over the course of the experiment. After an initial conditioning period during the first ∼100 cycles, the stroke stabilized at approximately 14 mm, corresponding to a contraction ratio of about 0.095. After this conditioning period, the contraction stroke varied by less than 3%. Although the absolute high- and low-pressure displacements shifted gradually during conditioning and prolonged cycling, no specimen exhibited rupture or leakage. Representative contraction and pressure waveforms from early and late cycles are compared in Fig. 9, showing that the waveform shape and contraction amplitude remain consistent over the full test.

These results demonstrate that the LT-PAM maintains a stable contraction stroke during prolonged repeated actuation, satisfying durability requirements for musculoskeletal robotics, repeated-actuation experiments, and assistive-motion studies.

**Table 2:** Steady-state displacement characteristics of the LT-PAM under repeated pressure cycling. Cycle 100 is used as the reference condition.

Cycle	High-pressure disp. [mm]			Low-pressure disp. [mm]			Stroke [mm]	Stroke change [%]
	Mean	SD	Change [%]	Mean	SD	Change [%]	Mean	
1	7.43	3.25	−28.05	21.14	2.77	−13.74	13.71	−3.33
100	10.32	4.17	0.00	24.50	3.60	0.00	14.18	0.00
4500	12.55	4.72	＋21.58	27.13	4.42	＋10.72	14.58	＋2.81
9000	13.22	4.91	＋28.15	27.83	4.53	＋13.58	14.61	＋2.98

### 7.3 Limitations

The LT-PAM is deliberately optimized for low-cost, reproducible in-house fabrication rather than for peak performance, and this entails several trade-offs. First, it is not intended to match high-end commercial actuators in absolute specifications such as maximum force density, positioning precision, or certified lifetime; its force and pressure ranges are bounded by the off-the-shelf silicone tube and crimp hardware. Second, because the actuator is hand fabricated, some sample-to-sample variability is unavoidable, in contrast to machine-manufactured commercial products. In the present study, all specimens were produced by a single fabricator, so we did not independently quantify sensitivity to fabrication tolerances or operator skill. However, the small number of parts and assembly steps, together with the elimination of skill-dependent adhesive curing through crimp-based fixation, should keep this sensitivity low, consistent with the small sample-to-sample standard deviations reported above. Third, applications that require tightly guaranteed unit-to-unit consistency — for example, deployment as a finished commercial or industrial product, or open-loop force control without per-unit calibration — are better served by commercial actuators. Similarly, applications requiring high dynamic bandwidth or certified long-term reliability fall outside the intended scope of this hardware and are left as future work.

## CRediT authorship contribution statement

**Keisuke Naniwa:** Writing – review & editing, Writing – original draft, Supervision, Conceptualization. **Yasuhiro Sugimoto:** Writing – review & editing, Investigation, Data curation. **Daisuke Nakanishi:** Writing – review & editing, Supervision, Project administration. **Yoichi Masuda:** Writing – review & editing, Methodology, Conceptualization.

## Ethics statements

This work did not involve human subjects or animal experiments.

## Declaration of generative AI and AI-assisted technologies in the manuscript preparation process

During the preparation of this manuscript, the authors used generative AI tools, including ChatGPT (OpenAI) and Claude (Anthropic, Claude Sonnet 4.6), for the following purposes: (1) literature-review support, (2) English-language refinement and editing, and (3) assistance in editing the English wording of figure captions and axis/label text. The AI tools were not used to generate or modify any figures, images, or the graphical content of the manuscript. All experimental procedures, hardware design decisions, data collection, analysis, and scientific interpretation were performed solely by the authors. The authors have reviewed all AI-assisted content and take full responsibility for the accuracy and integrity of this manuscript.

## Declaration of competing interest

The authors declare that they have no known competing financial interests or personal relationships that could have appeared to influence the work reported in this paper.
